# Genome-wide identification and expression profiles of ERF subfamily transcription factors in *Zea mays*

**DOI:** 10.7717/peerj.9551

**Published:** 2020-07-17

**Authors:** Lidong Hao, Shubing Shi, Haibin Guo, Ming Li, Pan Hu, Yadong Wei, Yanfei Feng

**Affiliations:** 1 College of Agriculture, Xinjiang Agriculture University, Urumqi, China; 2 College of Agriculture and Hydraulic Engineering, Sui Hua University, Suihua, China; 3 School of Economics and Management, Sui Hua University, Suihua, China

**Keywords:** Maize, ERF, Genome-wide, Expression profiles, Abiotic stress

## Abstract

The Ethylene-Response Factor (ERF) subfamily transcription factors (TFs) belong to the APETALA2/Ethylene-Responsive Factor (AP2/ERF) superfamily and play a vital role in plant growth and development. However, identification and analysis of the ERF subfamily genes in maize have not yet been performed at genome-wide level. In this study, a total of 76 ERF subfamily TFs were identified and were found to be unevenly distributed on the maize chromosomes. These maize ERF (ZmERF) TFs were classified into six groups, namely groups B1 to B6, based on phylogenetic analysis. Synteny analysis showed that 50, 54, and 58 of the *ZmERF* genes were orthologous to those in rice, Brachypodium, and Sorghum, respectively. *Cis*-element analysis showed that elements related to plant growth and development, hormones, and abiotic stress were identified in the promoter region of *ZmERF* genes. Expression profiles suggested that *ZmERF* genes might participate in plant development and in response to salinity and drought stresses. Our findings lay a foundation and provide clues for understanding the biological functions of ERF TFs in maize.

## Introduction

The Ethylene-Response Factor (ERF) subfamily transcription factors (TFs) belong to the APETALA2/Ethylene-Responsive Factor (AP2/ERF) superfamily, one of the largest groups of TFs in plants. Based on of repetitions and the sequence of the AP2 domain, the AP2/ERF TFs are classified into four separate subfamilies namely AP2, ERF, dehydration-responsive element-binding protein (DREB), and related to ABI3/VP1 (RAV) subfamilies (Nakano et al., 2006). The AP2 subfamily has two repeated AP2 domains, the ERF and DREB subfamilies contain a conserved WLG-motif and a single AP2 domain, while the RAV subfamily harbor a single AP2 domain in addition to a B3 DNA binding domain (Nakano et al., 2006). Although the ERF and DREB subfamilies only contain a single AP2 domain, there are some difference between them. DREB subfamily members are major factors involved in plant abiotic stress responses by regulating gene expression via the *cis*-acting DRE/C-repeat (DRE/CRT) element with a core motif of A/GCCGAC (Yamaguchi-Shinozaki & Shinozaki, 2006), while the ERF subfamily members binding to the ethylene-responsive (ERE) element AGCCGCC (Mizoi, Shinozaki & Yamaguchi-Shinozaki, 2012). The proteins of the ERF subfamily were classified into six groups termed B1 to B6 based on the phylogenetic relationship among them (Nakano et al., 2006).

Many of the ERF subfamily members are implicated in several diverse functions in plant development. For example, overexpression of Arabidopsis *ESR1* induces initiation of shoot regeneration (Banno et al., 2001) and Arabidopsis *LEP* is involved in cell division activity in the marginal meristem (Van der Graaff et al., 2000). The ERF subfamily members are also involved in response to several stress factors. For example, the overexpression of wheat *TaERF1* enhanced tolerance to stress induced by abscisic acid (ABA), ethylene, and salicylic acid (SA) (Xu et al., 2007); overexpression of barley *ERF2.11* in Arabidopsis enhances plant waterlogging tolerance (Luan et al., 2020); *ZmERF1* is activated and in response to drought stress via the ethylene and ABA signaling pathways (Shi et al., 2016); and the tobacco stress-induced gene 1 (*Tsi1*) is induced by salt and SA and overexpression of *Tsi1* improves tolerance to attacks from pathogens and osmotic stress (Park et al., 2001).

Until now, several ERF subfamily TFs have been identified in plants. For example, there are 77 in *Oryza sativa* (Sharoni et al., 2011), 65 in *Arabidopsis thaliana* (Sakuma et al., 2002), 91 in *Populus trichocarpa* (Zhuang et al., 2008), and 62 in *Glycine max* (Zhang et al., 2008), amongst others. However, no research has been performed on the identification and characterization of the ERF subfamily in maize. Maize (*Zea mays*) is one of the most important crops in the world. In this study, we conducted genome-wide identification of ERF subfamily TFs in maize and performed a phylogenetic relationship analysis. Exon-intron structures, conserved motifs, and expression patterns were also analyzed. This study establishes a foundation for further analysis of ERF subfamily TFs in maize and other plant species.

## Materials and Methods

### Identification of ERF TFs in the maize genome

The following steps were performed to identify the ERF TFs in maize. Firstly, the Hidden Markov Model (HMM) profile (PF00847) of the AP2 domain was downloaded from the Pfam v32.0 database (El-Gebali et al., 2019) and used to search against the maize protein sequences using a threshold of E<1e−5. Secondly, the putative maize ERF sequences were analyzed with the SMART program (Ivica & Peer, 2017) to confirm the presence of the AP2 domain. Proteins that contained two repeated AP2 domains and the B3 domain were eliminated. Since both the ERF and DREB subfamilies have only one AP2 domain, we aligned the ERF and DREB members by using the T-COFFEE method to order to differentiate them (Notredame, Higgins & Heringa, 2000). Members that did not contain an amino acid motif, AAEIRD, in the AP2 domains were eliminated (Sakuma et al., 2002). Finally, the putative ERF subfamily members were obtained.

The physicochemical properties, including isoelectric points (*pI*), molecular weights (WM), and Grand average of hydropathicity (GRAVY) of the ZmERFs were calculated by using the ExPASy (Gasteiger et al., 2003). The subcellular localization was predicted by CELLO v.2.5 software (Yu et al., 2006). The chromosome distribution and sequence information of ZmERFs and other plant species were downloaded from the Ensembl Plants database (Bolser et al., 2017).

### Sequence alignment, phylogenetic tree construction, and gene duplication analysis

Neighbor joining (NJ) and maximum likelihood (ML) trees were constructed using MEGA7 software with 1,000 bootstrap replications based on full-length protein sequences alignment (Sudhir, Glen & Koichiro, 2016). The trees were visualized online by using Evolview (https://www.evolgenius.info/evolview/). Segment duplication and tandem duplication pairs were elucidated from the Plant Genome Duplication Database (PGDD) (Lee et al., 2017). The collinear chart of *ERF* genes was drawn using the Circos program (Krzywinski et al., 2009).

### Gene structure, conserved motifs, and cis-elements analyses

The exon-intron structures of the *ZmERF* genes were constructed by GSDS v2.0 (Guo et al., 2007) using the CDS (coding sequences) and DNA sequences of the ZmERFs. The MEME Suite v5.1.1 (Bailey et al., 2015) was used to identify motifs within ZmERF TFs and the parameter as follows: the optimum motif width: 6-50; the maximum number of motifs: 10. The 2-kb upstream genomic DNA sequences of *ZmERF* genes were submitted to the PlantCARE database (Magali et al., 2002) to identify the *cis*-elements.

### Expression profiles

High-throughput sequencing data of maize in different plant tissues and under abiotic stresses were obtained from the Expression Atlas datasets of EMBL-EBI (Madeira et al., 2019) under accession number E-MTAB-3826 and the NCBI SRA database (Leinonen, Sugawara & Shumway, 2011) under SRP061276, respectively. These high-throughput data were used to analyze the expression of *ZmERF* genes in different tissues and under salinity and drought stresses. Based on the source of the high-throughput data, each tissue part had three biological replicates (Lunardon et al., 2016; Wang et al., 2016). The Fragments Per Kilobase Million (FPKM) values were used to calculate expression levels of the *ZmERF* genes and were visualized by the OmicShare tool online (http://www.omicshare.com/tools).

### Plant materials, salt treatment, and qRT-PCR

The maize cultivar “*Suiyu* 23” was planted in soil with a growth chamber at 24 ± 2 °C (16 h/8 h = light/dark). For salt and drought treatment, two-week seedlings were incubated in a Murashige and Skoog liquid medium containing 200 mM NaCl for salt stress or 20% polyethylene glycol (PEG) 6000 for drought stress for 0 (control), 3, 6, and 12 h, respectively, after which the whole seedlings were collected for RNA isolation. Every sample had two biological replicates and at least 20 whole seedlings were collected for each replicates. RNA extraction and cDNA synthesis were carried out according to the manufacturer instructions (TIANGEN Biotech CO,. LTD, Beijing). The qRT-PCR analyses were performed in triplicate for each of the biological replicates and the reaction mixtures contained 7.5 µl of 2 × Talent qPCR PreMix, 0.5 µl (10 µM) each of the forward and reverse primers, 0.5 µl of cDNA (200 ng/µl), 0.15 µl of 50 × ROX reference dye, and 5.85 µl ddH_2_O. The qRT-PCR reaction conditions were conducted according to the manufacturer instructions (TIANGEN Biotech CO,. LTD, Beijing). The qRT-PCR data acquisition and analyses were performed using QuantStudio™ Real-Time PCR Software (ThermoFisher Scientific). The relative expression level was computed by using the 2^−ΔΔCt^ analysis method (Livak & Schmittgen, 2000). Data were analyzed, and graphs were drawn using Microsoft Excel 2013. Standard deviations are indicated by error bars and significant differences are indicated with “*” (*P* < 0.05) or “**” (*P* < 0.01). Primers were designed by Oligo v7 software (Rychlik, 2007) and are listed in Table S1.

## Results

### Identification and classification of ZmERF TF subfamily in maize

To identify maize ERF TFs, a Hidden Markov Model (HMM) search was conducted using the HMM profile of the AP2 domain (PF00847) as a query against the maize genome protein sequences. A total of 191 putative ERF TFs were discovered in the maize genome. Subsequently, all putative TFs were determined to check the number of AP2 domains in the encoded proteins using the SMART and NCBI CDD program. Finally, a total of 157 genes contained a single or partial AP2 domains were obtained. To differentiate the DREB and ERF subfamily members, multiple sequence alignment was performed, and the conserved AP2 domains were obtained. As shown in Fig. S1, all DREB and ERF subfamily members were highly conserved in AP2/ERF regions. A pervious study showed that the amino acid motif, AAEIRD, is a clear characteristic of ERF subfamily members (Sakuma et al., 2002). Therefore, after sequence alignment, the putative ERFs that did not contain the AAEIRD motif in the AP2/ERF regions were eliminated. Thus, there were 76 encoded ERF TFs and 81 encoded DREB TFs.

**Figure 1:**
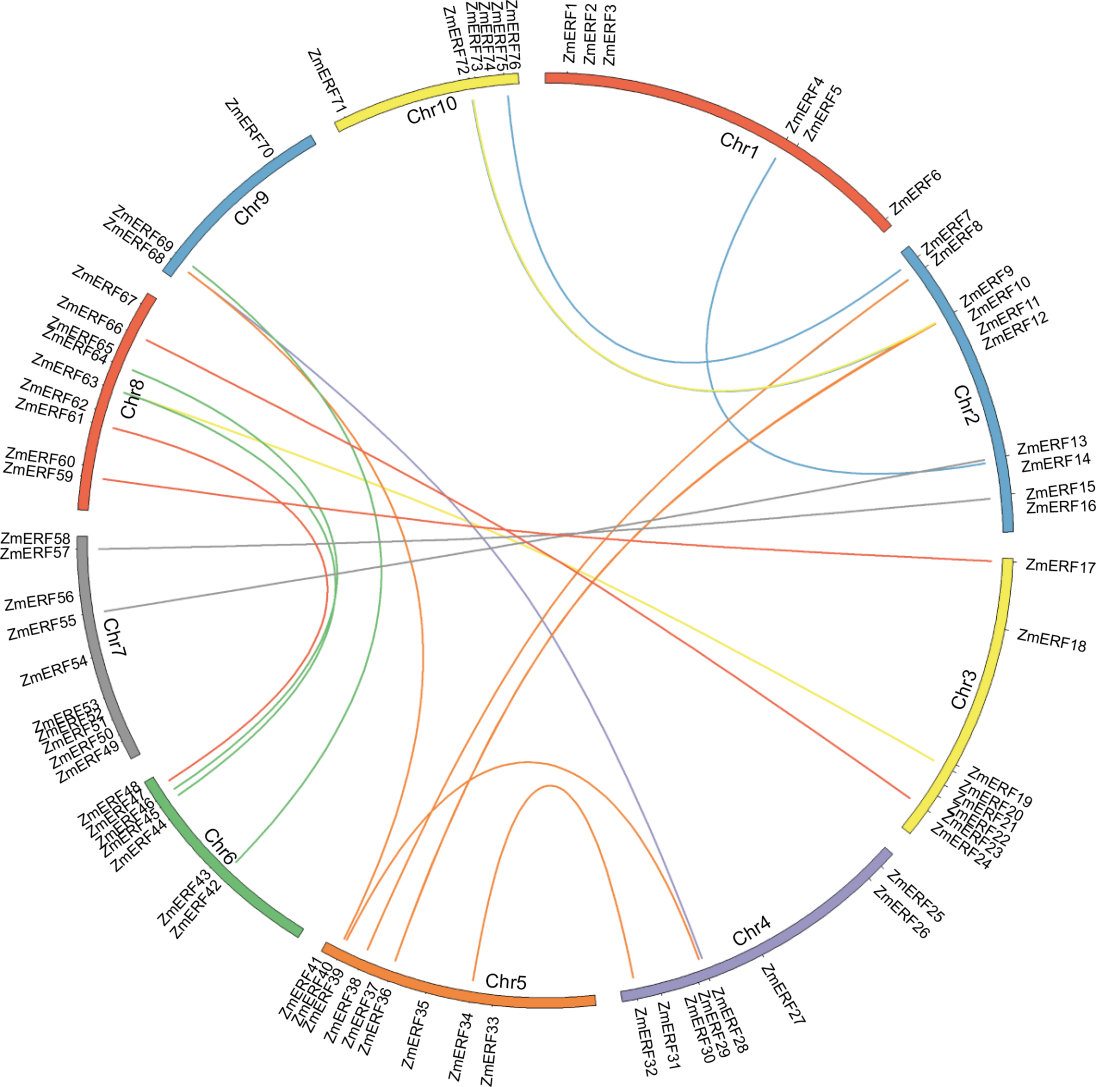
Chromosome location and duplication of ZmERF genes on maize chromosomes. Chromosomes of maize were colored in different color, and the connecting lines indicate duplicated gene pairs.

All CDS of ERF subfamily members were verified by Expressed Sequence Tags (ESTs) in the NCBI database (https://blast.ncbi.nlm.nih.gov/Blast.cgi). All predicted ZmERF TFs were named from ZmERF1 to ZmERF76 based on their chromosomal orders. They were unevenly distributed on the 10 chromosomes (Fig. 1). Chromosome (Chr) 4 and Chr 7 were the two largest chromosomes with 10 ZmERFs, while Chr 9 was smaller with 3 ZmERFs. The length of ZmERF proteins ranged from 183 (ZmERF44) to 1,425 amino acids (ZmERF24). The *pI* s ranged from 4.44 (ZmERF11) to 10.46 (ZmERF46). Subcellular location prediction was predicted online by CELLO and results indicated that most of them (71/76) were located in the nucleus. Detailed information on the 76 ZmERF TFs is listed in Table S2.

**Figure 2:**
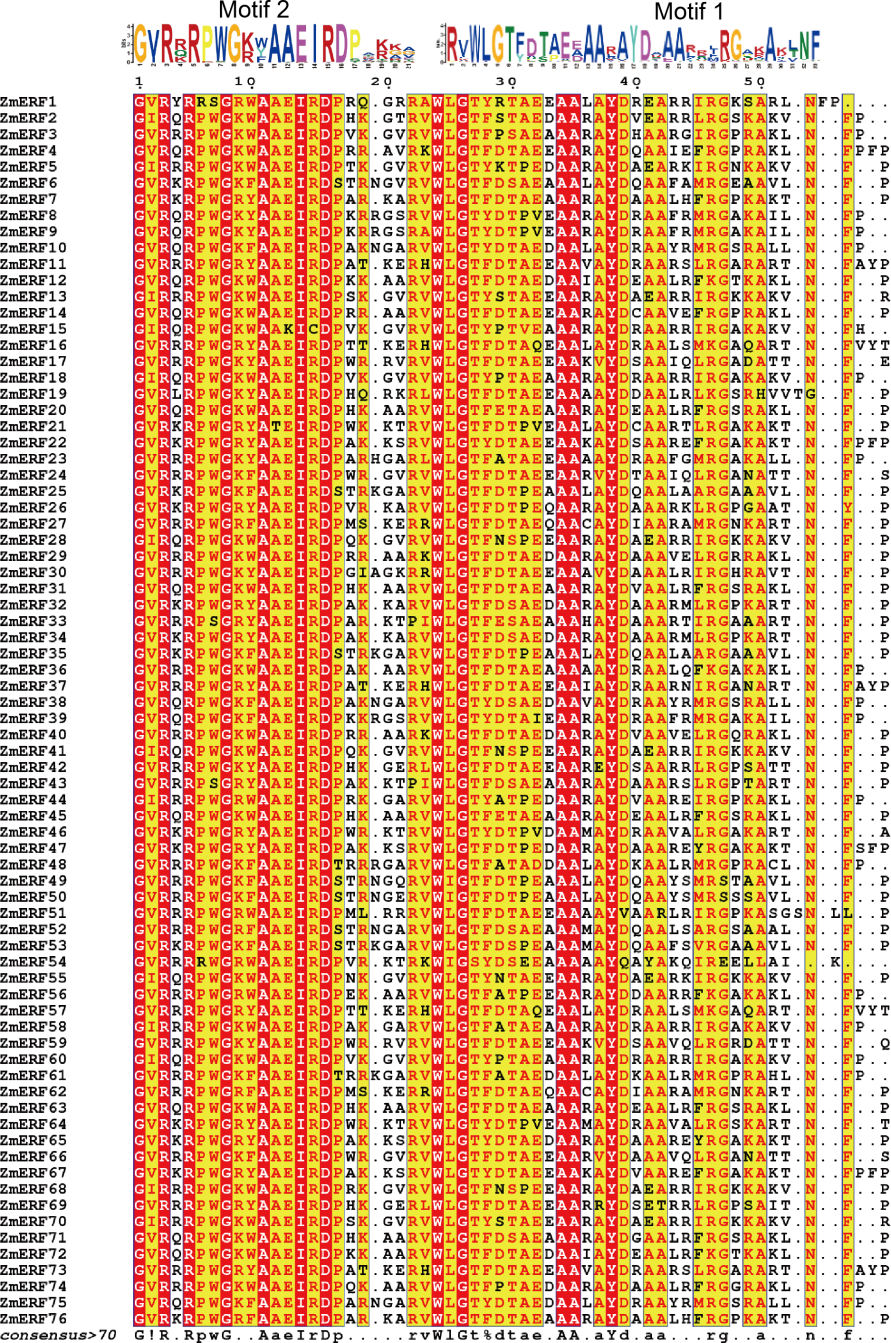
Sequence alignment of the AP2 domains in the 76 ZmERF TFs. Residues were marked by red indicated 100% conservation; motif 1 and motif 2 constituted the AP2 domain.

### Sequence alignment and phylogenetic relationship of ZmERFs

The AP2 domain is a gene structural feature in plant ERFs and DREBs, and each ERF and DREB member contain a single AP2 domain. To further analyze maize ERF subfamily TFs, the full-length protein sequences of ZmERFs were aligned by using the T-COFFEE method (Notredame, Higgins & Heringa, 2000). Results showed that AP2 domains were highly conserved in the maize ERF subfamily TFs (Fig. 2). There were invariant amino acids in the AP2 domains, including three glycine residues (G), two arginine residues (R), three alanine residues (A), one isoleucine residue (I), one aspartic residue (D), one tryptophan residue (W), and one tyrosine residue (Y), and they were 100% conserved inside the AP2 domains of maize ERF subfamily members. We also aligned the maize ERF and DREB subfamily members. Only three residues (G-26, A-34, and A-35) were 100% conserved in the AP2 domains (Fig. S1). Based on corresponding alignment, a stringent motif (GVR[RQK]RPWG[KR][WYF]AAEIRDPA[KR][KG][AGV]) was recognized in the ZmERF subfamily members. Multiple sequences of 13 known ERF subfamily genes also showed that the AAEIRD motif is highly conserved in the AP2 domains (Fig. S2), further indicated that the AAEIRD motif is the signature of ERF subfamily members. Moreover, sequence alignment showed that there were 14 ZmERF TFs (ZmERF7, -21, -22, -26, -32, -33, -34, -46, -47, -58, -63, -64, -65, and -76) that contained the ERF-associated amphiphilic repression (EAR) motif [(L/F)DLN(L/F)xP], which is essential for ERF TFs to repress ethylene biosynthesis (Zhang et al., 2013).

**Figure 3:**
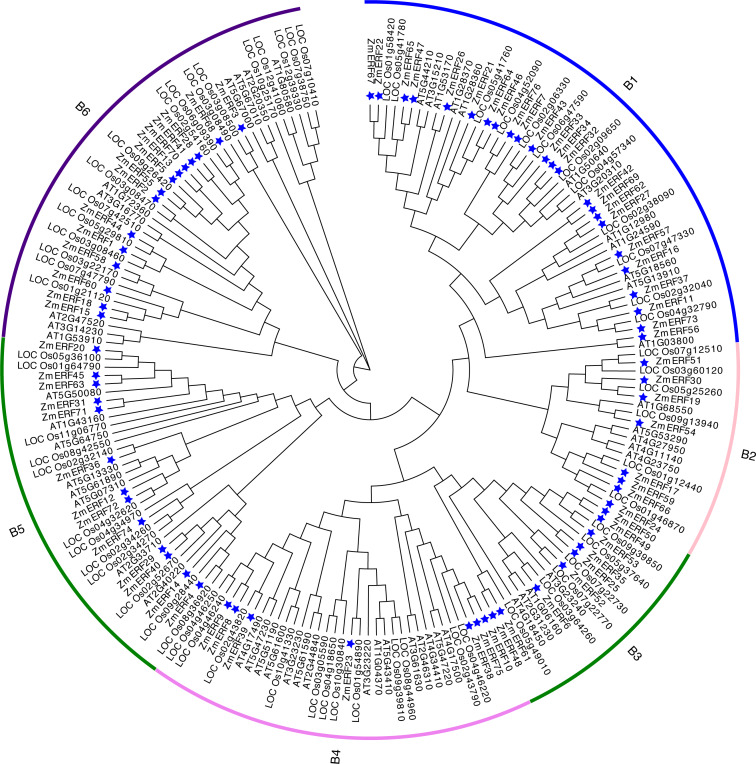
Neighbor-joining (NJ) tree of *Arabidopsis*, rice, and maize ERF subfamily members. The blue stars indicate the maize *ERF* genes.

**Figure 4:**
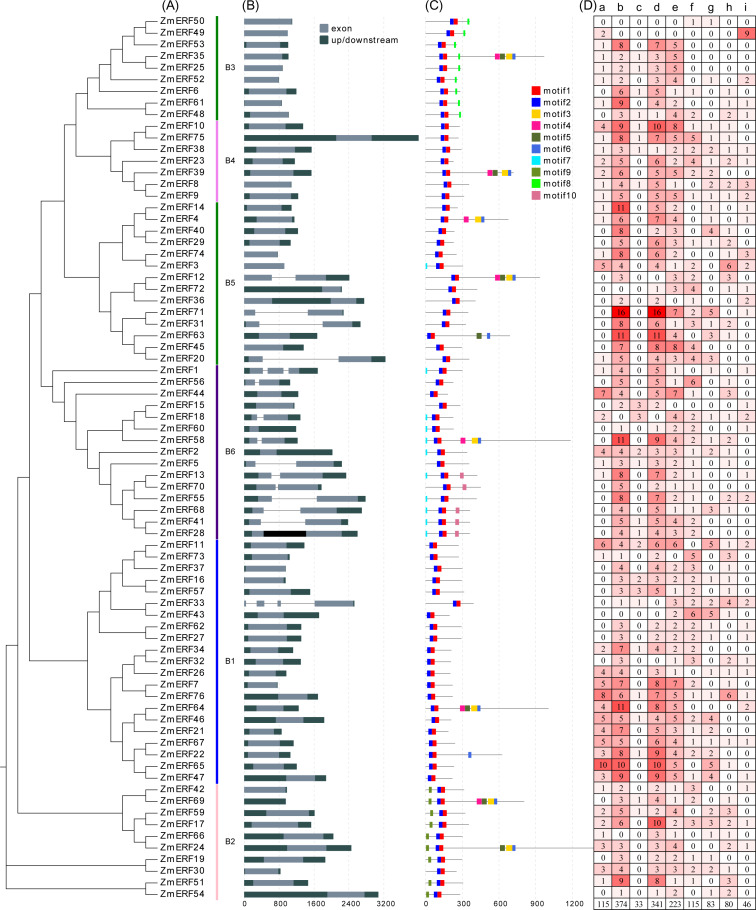
(A) Phylogenetic relationships, (B) gene structures, (C) conserved motifs, and (D) *cis*-elements of ZmERFs. In (A) maize ERF subfamily members are classified into six subgroups; (B) exons and introns were indicated by light dark grey boxes and dark grey lines, respectively, and UTRs were indicated by grey boxes; (C) conserved motifs were displayed in different colors; (D) lowecase letters a-i indicate Sp1, G-Box, O2-site, ABRE, as-1, ARE, DRE core, LTR, MBS *cis*-elements in the promoter of *ZmERF* genes.

To understand the evolutionary relationship of ERF TFs in Arabidopsis, rice, and maize, un-rooted NJ and ML phylogenetic trees were constructed based on the alignment of ERFs. The NJ tree (Fig. 3) and the ML tree (Fig. S3) were consistent. We classified these ZmERFs into six groups that were named B1-B6 based on the bootstrap values. Additionally, an un-rooted NJ phylogenetic tree of ZmERF TFs was also constructed (Fig. 4A). Results showed that group B1 was the largest group with 21 members, while group B4 was the smallest with 7 members.

### Gene structure and conserved motif analyses

Gene structure can help to understand *ERF* gene evolution. As shown in Fig. 4B, the number of exons of *ZmERF* genes varied from one to four. Except for 15 *ZmERF* genes with 2 or more exons, other members only had one exon. In general, members of the same family might share similar gene structures. For example, all group B2, B3, and B4 members contained only one exon, and most group B6 members had two exons.

We further predicted the conserved motifs of ZmERF proteins. As shown in Fig. 4C, 10 motifs were identified. All ZmERF proteins had motif 1 and motif 2, and these two motifs constituted the AP2 domain (Figs 2 and 4C). Motif 7 and 9 were only found in group B2, while motif 8 was only identified in group B3. The consensus sequences of these motifs are given in: Table S3.

### Gene duplication analysis

In terms of gene duplication, no tandem duplication was observed, and 37 ZmERFs (48.68%) formed 21 segmental duplication pairs that were located on 10 chromosomes (Fig. 1 and Table S4). The synteny relationship between ZmERFs and other plant species was also investigated. As shown in Tables S4 and S5, results showed that there were 0, 50, 54, and 58 *ZmERF* genes that showed a syntenic bias towards particular Arabidopsis, rice, Brachypodium, and Sorghum chromosomes, respectively (Table S5). The substitution rate (non-synonymous/synonymous, Ka/Ks) is an effective index to evaluate the positive selection pressure after duplication, the Ka/Ks value of each gene pair was less than 1, indicated that these genes had undergone purifying selection, which further implied that the ERFs of plants from the gramineae family had strong phylogenetic relationship.

### *Cis*-element analysis

The *cis*-elements of promoter regions are related to the regulation of the gene expression patterns and functions, and they are recognized by TFs that recruit the transcriptional machinery (Todeschini, Georges & Veitia, 2014). In this study, *cis*-elements in the 2-kb promoter regions of *ZmERF* genes were searched by using the PlantCARE web tool (Magali et al., 2002). As results (Fig. 4D), three kinds of *cis*-elements, plant growth and development-related, and hormones-related, and stresses-related, were identified. The *cis*-elements related to plant growth and development include the light-responsive element Sp1 (GGGCGG) (Haidar, Dale & Harris, 1991) and G-box (CACGTC) (Giuliano et al., 1988), and metabolism regulation related element O2-site (Carlini et al., 1999). The *cis*-elements related to hormones and abiotic stress include the drought-inducibility element MBS (CAACTG) (Zhang et al., 2015), the ABA-responsive element ABRE (ACGTG) (Ezcurra et al., 1999), the low-temperature responsiveness element LTR (CCGAAA) (Dunn et al., 1998), and the anaerobic induction element ARE (AAACCA) (Geffers et al., 2001). Among these *cis*-elements, the G-Box and ABRE were the two most frequently identified *cis*-elements. In combination with the phylogenetic tree, these results show that the phylogenetically similar genes share identical *cis*-elements. For example, group B3 members *ZmERF25*, -*35*, and -*52* had the same proportion of *cis*-elements, while group B1 members *ZmERF27* and *ZmERF62* harbored the same proportion of *cis*-elements.

**Figure 5:**
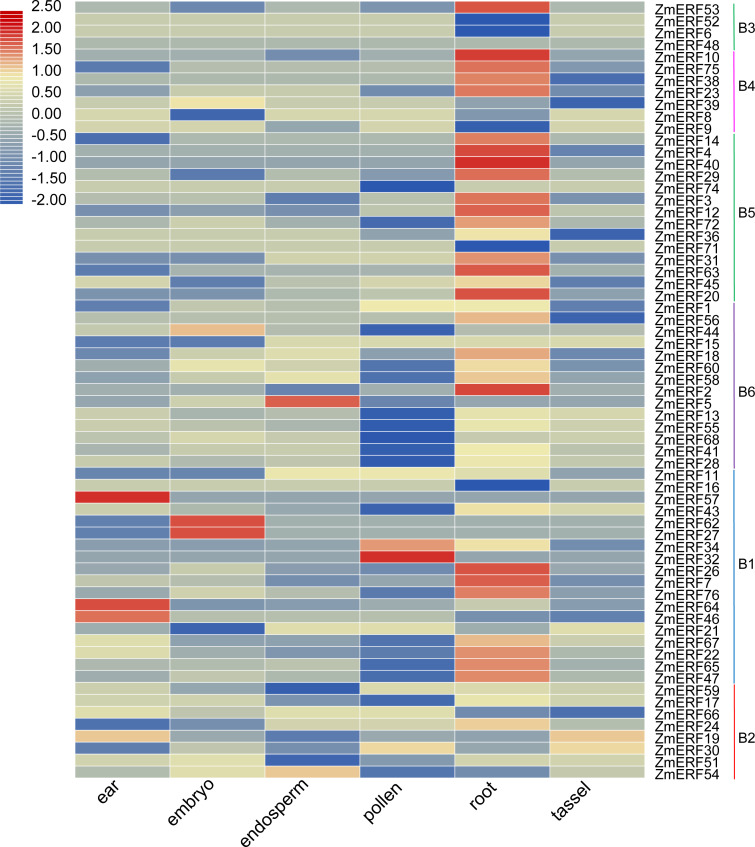
Expression profiles of ZmERFs in six tissues. Transcriptome data was used to investigate expression profiles of *ZmERF* genes. The colour scale represents RPKM which were normalized log10 transformed counts. Blue indicates low expression and red indicates high expression.

### Expression profiles of ZmERF genes

We also investigated the tissue-specific expression levels of *ZmERF* genes in six different maize tissues based on a previous study (Wang et al., 2016). The RPKM values of *ZmERF* genes below 1 were considered as not expressed. As shown in Fig. 5, forty-three *ZmERF* genes were highly expressed in root tissues in comparison to other tissues, implying that they play a particular role in root development. Moreover, five genes (including group B1 members *ZmERF19* and *-59*, group B2 members *ZmERF46*, -*57* , and -*64*) were highly expressed in ear tissue, eight (*ZmERF15*, -*27*, -*39*, -*44*, -*51*, -*62*, -*66*, and -*68*) in embryo tissues, three (*ZmERF5*, -*9*, and -*54*) in endosperm tissue, and five (*ZmERF1*, -30, -32, -34, and -74) in the pollen. The expression of the same group shared similar expression. For example, most of group B3, B4, and B5 members were found to be specifically expressed in root tissue; many *ZmERF* genes (12/14) in group B6 had low expression level or not expressed in the pollen tissue. These results suggest that these genes might have important roles in plant growth and development.

**Figure 6:**
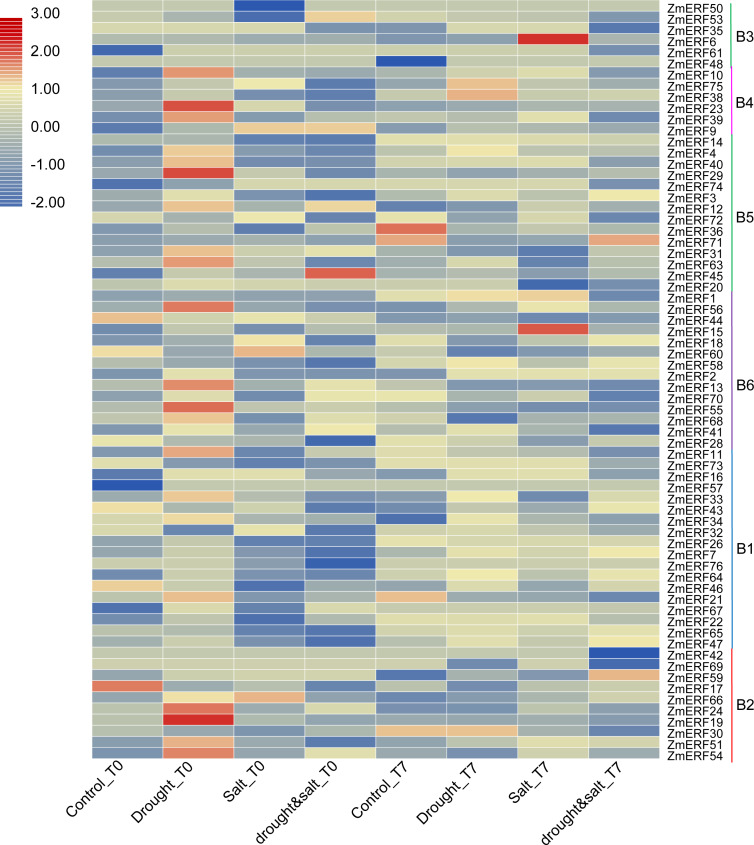
Expression profiles of ZmERFs under drought, salt, drought and salt combination treatments. Transcriptome data was used to investigate expression profiles of *ZmERF* genes. The colour scale represents RPKM which were normalized log10 transformed counts. Blue indicates low expression and red indicates high expression. T0 and T7 indicate ten days of treatment and seven days of post-treatment recovery respectively.

We also investigated their expression patterns by using the high-throughput data of maize under drought, salt, and drought and salt combination treatment on the basis of previous study (Lunardon et al., 2016). As a result, all of the *ZmERF* genes were detected (Fig. 6). Many genes were identified as differentially expressed genes by using the fold change method (log10-bias ratio) with more than two folds as criterion. After 10 days of growth under drought, salt, and drought and salt combination stress (T0), we characterized 22 (21 up-regulated, 1 down-regulated), 11 (4 up-regulated, 7 down-regulated), and 13 (6 up-regulated, 7 down-regulated), differentially expressed *ERF* genes were characterized, respectively (Table S6). In addition, 9 (7 up-regulated, 2 down-regulated), 31 (16 up-regulated, 15 down-regulated), and 38 (18 up-regulated, 20 down-regulated) genes were identified under drought, salt, and drought and salt combination stress, respectively, after seven days of post-treatment recovery (T7) (Table S6). In total, 56 differentially expressed genes were identified and only one *ZmERF* gene (*ZmERF31*) was commonly differentially expressed under all three stress condition for ten days (T0), and five (*ZmERF9*, -*33*, -*38*, -*63*, and -*72*) were commonly differentially expressed after seven days of post-treatment recovery (T7). Differentially expressed genes in the same group showed similar expression profiles at some level. For example, 10 of 11 *ZmERF* genes (except for ZmERF72) in group B5 were up-regulated after 10 days of growth under drought stress (Drought_T0); 3 of 4 genes in group B4 and 5 of 10 genes in group B5 were up-regulated under salt stress after seven days of post-treatment recovery (Salt_T7); majority (7/10) of group B1 genes were down-regulated under drought stress after seven days of post-treatment recovery (drought&salt_T7). However, no simultaneously differentially expressed genes were found under these six treatments, which indicates the complicated influence of drought and salt on plants, and further suggests that the expression levels of *ZmERF* genes were altered by different stress.

**Figure 7:**
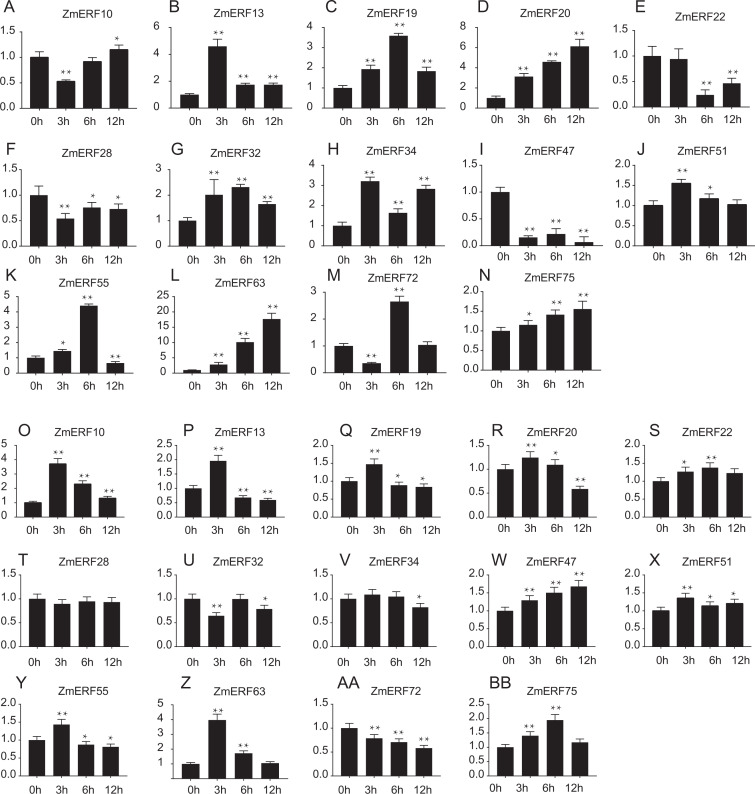
Expression patterns of 14 selected *ZmERF* genes under (A-N) salt and (O-AB) drought treatment. The horizontal axe indicates different time points in 0 (control), 3, 6, and 12 h after (A–N) salt or (O–BB) drought treatment, the verticals axe stands for the relative expression level. Error bars represent standard deviations, and statistically significant differences are indicated: *, *P* < 0.05; **, *P* < 0.01 (Student’s *t*-test).

Furthermore, we performed qRT-PCR to analyze the expression patterns of 14 randomly selected *ZmERF* genes under salt and drought treatment. Results showed that all of them were induced by 200 mM NaCl and 20%PEG6000 (Fig. 7). Under salt treatment (Figs. 7A–7N), the expression levels of *ZmERF13*, -*34*, -*51* increased after 3 h and then decreased slightly compared to control (0 h); the expression levels of *ZmERF19*, -*32*, and -*55* were gradually up-regulated and finally decreased at 12 h; and the expression levels of *ZmERF20*, -*63*, and -*75* were gradually up-regulated and finally peaked at 12 h. However, *ZmERF22*, -*28*, and -*47* were down-regulated after salinity treatment. Using the fold change method (log10-bias ratio) with more than two folds as criterion, some of the *ZmERF* genes were differentially expressed. For example, *ZmERF10*, -*13*, -*20*, -*34*, -*47*, -*63*, and -*72* under salt stress for 3 h. Under drought treatment (Figs. 7O–7AB), the expression levels of *ZmERF10*, -*13*, -*19*, -*20*, -*55*, and -*63* increased after 3 h compared to 0 h, and *ZmERF75* increased at 6 h. *ZmERF47* was gradually up-regulated while *ZmERF72* was down-regulated after drought treatment. However, the expression levels of *ZmERF28* and -*32* were not significantly regulated after drought stress. There were only two genes, *ZmERF10* and -*63*, that were differentially expressed under drought stress for 3 h. Compared to other time-points, the expression levels of 3 h under salt and drought stress were significantly altered.

## Discussion

### The characteristics of ERF subfamily TFs

The ERF and DREB subfamily TFs are characterized by a single AP2 region. However, little research has been conducted on ERF subfamily in maize. In this study, a total of 76 ZmERF TFs were identified after searched against maize genome protein sequences and all of them were validated by ESTs in the NCBI database. Phylogenetic analyses revealed that the ZmERFs were classified into six groups, with group B1 being the largest with 21 members, and group B4 was the smallest with 7 members. In combination with the phylogenetic tree, the phylogenetically similar ZmERF shared similar gene structures, conserved motifs, and *cis*-elements.

Compared to DREB subfamily members, there is a clear characteristic of maize ERF subfamily members in the region corresponding to the second β-strand, which is an AAEIRD motif. A pervious study has shown that the AAEIRD motif only occurs in the Arabidopsis ERF subfamily members (Sakuma et al., 2002). In this study, all maize ERFs and 13 known plant ERF subfamily members contained a single AP2 domain and all of them contained an AAEIRD motif within the AP2 domain, further confirmed that the AAEIRD motif is conserved in plant ERF subfamily members. The conserved motif analysis showed that the motif pattern (GVR[RQK]RPWG[KR][WYF]AAEIRDPA[KR][KG][AGV]) was the AP2 domain among these 76 ZmERFs.

Gene duplication can lead to generate a large number of novel genes (Li et al., 2013). In this study, no tandem duplication was found and 21 segmental duplication pairs were identified in the maize genome. Also, the Ka/Ks of all gene pairs were less than 1, suggesting that these genes had evolved under strong purifying selection. Synteny analysis also showed that the *ERF* genes of monocots have strong synteny relationships.

### The functions of ERF subfamily members

The EAR motif [(L/F)DLN(L/F)xP] is specifically identified in most plant ERF subfamily members and found to involved in repression function of these TFs (Nakano et al., 2006; Zhao et al., 2019b). In plants, the EAR motif of wheat Q protein and rice OsERF3 protein are found to repress transcriptional activity (Liu et al., 2018; Xie et al., 2018; Zhang et al., 2013). In this study, there were 17 ZmERF TFs that contained the EAR motif, indicating that they might be essential for transcriptional repression activity of target proteins.

Studies also showed that many ERF subfamily TFs bind to the GCC-boxes (GCCGCC) and are involved in hormone responses (Fujimoto et al., 2000; Mantiri et al., 2008; Oñate Sánchez & Singh, 2002). For example, rice OsERF922 binds to the GCC-box and acts as a transcriptional activator in plant cells (Liu et al., 2012). In maize, *ERF* gene *branched silkless1* (*ZmERF57* in this study) regulates meristem identity from lateral domains of the spikelet meristem (Chuck et al., 2002) and maize *ERF1* (*ZmERF60* in this study) in response to hormones via ethylene and ABA signaling pathways (Shi et al., 2016). In this study, genes in the same group shared similar expression. For example, most of the group B3, B4, and B5 members were found to be specifically expressed in root tissue; two group B1 members ZmERF19 and -59 and B2 members ZmERF46, -57, and -64 were highly expressed in ear tissue; group B1 members ZmERF32 and -34 showed high expression level in the pollen tissue, these results implying that they play a particular role in plant development, further indicating that their functions are conservative to some extent. *Cis*-element analyses demonstrated that *cis*-elements related to hormone and abiotic stress are found in the promoter of *ZmERF* genes, further suggesting the probable function of *ZmERF* genes in response to environmental stress.

Most ERF subfamily TFs facilitate tolerance against environmental stress, such as drought, high salinity, and extreme temperatures (Fujimoto et al., 2000; Park et al., 2001). For example, soybean *GmERF135* was extremely up-regulated by drought, overexpression of *GmERF135* in Arabidopsis enhanced tolerance to drought condition (Zhao et al., 2019a); and overexpression of rice *OsERF922* exhibited decreased tolerance to salt stress (Liu et al., 2012). Consistent with *GmERF135* and *OsERF922*, the homology genes *ZmERF39* and *ZmERF23* were also up-regulated by drought and salt stress, respectively, indicating that they have a function important role in drought and salt tolerance. Genome-wide expression analysis of AP2/ERF family genes in rice, soybean, tomato, and poplar reveal that many ERF TFs are induced by drought and high salinity (Oñate Sánchez & Singh, 2002; Sharoni et al., 2011; Zhang et al., 2008; Zhuang et al., 2008). In this study, high-throughput data showed that most *ZmERF* genes were expressed in response to drought and salt stress, 56 differentially expressed genes were identified among these stresses, and differently expressed genes in the same group had similar expression profiles at some level. For example, most ZmERFs in group B5 were up-regulated after 10 days of growth under drought stress (Drought_T0), majority (7/10) genes in the group B1 were down-regulated under drought stress after seven days of post-treatment recovery (drought&salt_T7). In addition, qRT-PCR analysis showed that *ZmERF* genes were induced by salinity and drought stress, and most of them were significantly regulated. For example, there were seven (*ZmERF10*, -*13*, -*20*, -*34*, -*47*, -*63*, and -*72*) and 2 (*ZmERF10* and -*63*) genes that were differentially expressed and significantly regulated under salt and drought stress for 3 h, respectively. These results suggest that these genes may play important roles in response to abiotic stress, further suggesting that the expression levels of *ZmERF* genes were altered by stress.

## Conclusions

A total of 76 ZmERF TFs were identified in the maize genome and divided into six groups. The same group members shared similar exon-intron structure and conserved motifs, most genes in the same group had similar expression patterns and shared identical *cis*-elements. Each ZmERF TF contained a single AP2 domain and this domain was characterized by an amino acid motif AAEIRD. Segmental duplication contributed to the expansion of ZmERFs, and these duplication pair genes had evolved under strong purifying selection. The *cis*-elements analysis suggested that the expression of ZmERFs was regulated by hormones and various environmental factors. High-throughput data analysis revealed that *ZmERF* genes were specifically expressed in different tissues and differentially expressed in different abiotic stresses. The expression profiles under salt and drought stresses by qRT-PCR analysis showed that some ZmERFs were significantly regulated, indicated their vital roles in response to salt and drought stresses. Our results provide a reference for further study of maize ERF subfamily genes.

##  Supplemental Information

10.7717/peerj.9551/supp-1 File S1 Raw data All plant sequences and RT-PCR raw data used in this study.

10.7717/peerj.9551/supp-2 Supplemental Information 2 Tables S1-S6 Table S1: The primers used in this study Table S2: The detailed information of ZmERF TFs Table S3: Motif patterns of maize ERF subfamily members Table S4: Gene segmental duplication of maize ERFs Table S5: Synteny analysis of ERFs between maize and other plants Table S6: Differentially expressed genes were identified under abiotic stresses (red indicat up-regulated, blue indicates down-regulated).

10.7717/peerj.9551/supp-3 Table S1 Multiple sequence alignment of maize ERF and DREB subfamily members

10.7717/peerj.9551/supp-4 Figure S2 Multiple sequence alignment of 13 known plant ERF subfamily members There is a clear AAEIRD-motif in the AP2 domain.

10.7717/peerj.9551/supp-5 Figure S3 Maximum likelihood (ML) tree of *Arabidopsis*, rice, and maize ERF subfamily members

## Additional Information and Declarations

### Data Availability

The following information was supplied regarding data availability: The raw measurements are available in the Supplemental Files.

## References

[ref-1] Bailey TL, Johnson J, Grant CE, Noble WS (2015). The MEME suite. Nucleic Acids Research.

[ref-2] Banno H, Ikeda Y, Niu Q-W, Chua N-H (2001). Overexpression of arabidopsis ESR1 induces initiation of shoot regeneration. The Plant Cell.

[ref-3] Bolser DM, Staines DM, Perry E, Kersey PJ (2017). Ensembl plants: integrating tools for visualizing, mining, and analyzing plant genomic data. Methods in Molecular Biology.

[ref-4] Carlini LE, Ketudat M, Parsons RL, Prabhakar S, Schmidt RJ, Guiltinan MJ (1999). The maize EmBP-1 orthologue differentially regulates Opaque2-dependent gene expression in yeast and cultured maize endosperm cells. Plant Molecular Biology.

[ref-5] Chuck G, Muszynski M, Kellogg E, Hake S, Schmidt RJ (2002). The control of spikelet meristem identity by the branched silkless1 gene in maize. Science.

[ref-6] Dunn MA, White AJ, Vural S, Hughes MA (1998). Identification of promoter elements in a low-temperature-responsive gene (blt4.9) from barley (Hordeum vulgare L.). Plant Molecular Biology.

[ref-7] El-Gebali S, Mistry J, Bateman A, Eddy SR, Luciani A, Potter SC, Qureshi M, Richardson LJ, Salazar GA, Smart A, Sonnhammer ELL, Hirsh L, Paladin L, Piovesan D, Tosatto SCE, Finn RD (2019). The Pfam protein families database in 2019. Nucleic Acids Research.

[ref-8] Ezcurra I, Ellerström M, Wycliffe P, Stålberg K, Rask L (1999). Interaction between composite elements in the napA promoter: both the B-box ABA-responsive complex and the RY/G complex are necessary for seed-specific expression. Plant Molecular Biology.

[ref-9] Fujimoto SYM, Ohta M, Usui A, Shinshi H, Ohme-Takagi M (2000). Arabidopsis ethylene-responsive element binding factors act as transcriptional activators or repressors of GCC box-mediated gene expression. The Plant Cell.

[ref-10] Gasteiger E, Gattiker A, Hoogland C, Ivanyi I, Appel RD, Bairoch A (2003). ExPASy: the proteomics server for in-depth protein knowledge and analysis. Nucleic Acids Research.

[ref-11] Geffers R, Sell S, Cerff R, Hehl R (2001). The TATA box and a Myb binding site are essential for anaerobic expression of a maize GapC4 minimal promoter in tobacco. Biochimica et Biophysica Acta.

[ref-12] Giuliano G, Pichersky E, Malik VS, Timko MP, Scolnik PA, Cashmore AR (1988). An evolutionarily conserved protein binding sequence upstream of a plant light-regulated gene. Proceedings of the National Academy of Sciences of the United States of America.

[ref-13] Guo AY, Zhu QH, Chen X, Luo JC (2007). GSDS: a gene structure display server. Hereditas.

[ref-14] Haidar MA, Dale H, Harris B (1991). Sp1 is essential and its position is important for p120 gene transcription: a 35 bp juxtaposed positive regulatory element enhances transcription 2.5 fold. Nucleic Acids Research.

[ref-15] Ivica L, Peer B (2017). 20 years of the SMART protein domain annotation resource. Nucleic Acids Research.

[ref-16] Krzywinski M, Schein J, Birol I, Connors J, Gascoyne R, Horsman D, Jones SJ, Marra MA (2009). Circos: an information aesthetic for comparative genomics. Genome Research.

[ref-17] Lee TH, Kim J, Robertson JS, Paterson AH (2017). Plant genome duplication database. Methods in Molecular Biology.

[ref-18] Leinonen R, Sugawara H, Shumway M (2011). The sequence read archive. Nucleic Acids Research.

[ref-19] Li M-Y, Wang F, Jiang Q, Li R, Ma J, Xiong A-S (2013). Genome-wide analysis of the distribution of AP2/ERF transcription factors reveals duplication and elucidates their potential function in chinese cabbage (Brassica rapassp.pekinensis). Plant Molecular Biology Reporter.

[ref-20] Liu D, Chen X, Liu J, Ye J, Guo Z (2012). The rice ERF transcription factor OsERF922 negatively regulates resistance to Magnaporthe oryzae and salt tolerance. Journal of Experimental Botany.

[ref-21] Liu P, Liu J, Dong H, Sun J (2018). Functional regulation of Q by microRNA172 and transcriptional co-repressor TOPLESS in controlling bread wheat spikelet density. Plant Biotechnology Journal.

[ref-22] Livak K, Schmittgen T (2000). Analysis of relative gene expression data using real-time quantitative PCR and the 2-ΔΔCt method. Methods.

[ref-23] Luan H, Guo B, Shen H, Pan Y, Hong Y, Lv C, Xu R (2020). Overexpression of barley transcription factor HvERF2.11 in arabidopsis enhances plant waterlogging tolerance. International Journal Molecular Science.

[ref-24] Lunardon A, Forestan C, Farinati S, Axtell MJ, Varotto S (2016). Genome-wide characterization of maize small RNA loci and their regulation in the required to maintain repression6-1 (rmr6-1) mutant and long-term abiotic stresses. Plant Physiology.

[ref-25] Madeira F, Park YM, Lee J, Buso N, Gur T, Madhusoodanan N, Basutkar P, Tivey ARN, Potter SC, Finn RD, Lopez R (2019). The EMBL-EBI search and sequence analysis tools APIs in 2019. Nucleic Acids Research.

[ref-26] Magali L, Patrice D, Gert T, Kathleen M, Yves M, Yves VDP, Pierre RRS (2002). PlantCARE, a database of plant cis-acting regulatory elements and a portal to tools for *in silico* analysis of promoter sequences. Nucleic Acids Research.

[ref-27] Mantiri FR, Kurdyukov S, Lohar DP, Sharopova N, Saeed NA, Wang X-D, VandenBosch KA, Rose RJ (2008). The transcription factor MtSERF1 of the ERF subfamily identified by transcriptional profiling is required for somatic embryogenesis induced by auxin plus cytokinin in medicago truncatula. Plant Physiology.

[ref-28] Mizoi J, Shinozaki K, Yamaguchi-Shinozaki K (2012). AP2/ERF family transcription factors in plant abiotic stress responses. Biochimica et Biophysica Acta.

[ref-29] Nakano T, Suzuki K, Fujimur T, Shinshi H (2006). Genome-wide analysis of the erf gene family in arabidopsis and rice. Plant Physiology.

[ref-30] Notredame C, Higgins DG, Heringa J (2000). T-coffee: a novel method for fast and accurate multiple sequence alignment. Journal of molecular biology.

[ref-31] Oñate Sánchez L, Singh KB (2002). Identification of arabidopsis ethylene-responsive element binding factors with distinct induction kinetics after pathogen infection. Plant Physiology.

[ref-32] Park JM, Park CJ, Lee SB, Ham BK, Shin R, Paek KH (2001). Overexpression of the tobacco Tsi1 gene encoding an EREBP/AP2-type transcription factor enhances resistance against pathogen attack and osmotic stress in tobacco. The Plant Cell.

[ref-33] Rychlik W (2007). OLIGO 7 primer analysis software. Methods in Molecular Biology.

[ref-34] Sakuma Y, Liu Q, Dubouzet JG, Abe H, Shinozaki K, Yamaguchi-Shinozaki K (2002). DNA-binding specificity of the ERF/AP2 domain of arabidopsis DREBs. Transcription factors involved in dehydration- and cold-inducible gene expression. Biochemical & Biophysical Research Communications.

[ref-35] Sharoni AM, Nuruzzaman M, Satoh K, Shimizu T, Kondoh H, Sasaya T, Choi I-R, Omura T, Kikuchi S (2011). Gene structures, classification and expression models of the AP2/EREBP transcription factor family in rice. Plant & Cell Physiology.

[ref-36] Shi Q, Dong Y, Zhou Q, Qiao D, Ma Z, Zhang L, Li Y (2016). Characterization of a maize ERF gene, ZmERF1, in hormone and stress responses. Acta Physiologiae Plantarum.

[ref-37] Sudhir K, Glen S, Koichiro T (2016). MEGA7: molecular evolutionary genetics analysis version 7.0 for bigger datasets. Molecular Biology & Evolution.

[ref-38] Todeschini AL, Georges A, Veitia RA (2014). Transcription factors: specific DNA binding and specific gene regulation. Trends in Genetics.

[ref-39] Van der Graaff E, Dulk-Ras AD, Hooykaas PJ, Keller B (2000). Activation tagging of the LEAFY PETIOLE gene affects leaf petiole development in Arabidopsis thaliana. Development.

[ref-40] Wang B, Tseng E, Regulski M, Clark TA, Hon T, Jiao Y, Lu Z, Olson A, Stein JC, Ware D (2016). Unveiling the complexity of the maize transcriptome by single-molecule long-read sequencing. Nature Communication.

[ref-41] Xie Q, Li N, Yang Y, Lv Y, Yao H, Wei R, Sparkes DL, Ma Z (2018). Pleiotropic effects of the wheat domestication gene Q on yield and grain morphology. Planta.

[ref-42] Xu ZS, Xia L-Q, Chen M, Cheng X-G, Zhang R-Y, Li L-C, Zhao Y-X, Lu Y, Ni Z-Y, Liu L (2007). Isolation and molecular characterization of the Triticum aestivum L. ethylene-responsive factor 1 (TaERF1) that increases multiple stress tolerance. Plant Molecular Biology.

[ref-43] Yamaguchi-Shinozaki K, Shinozaki K (2006). Transcriptional regulatory networks in cellular responses and tolerance to dehydration and cold stresses. Annual Review of Plant Biology.

[ref-44] Yu CS, Chen YC, Lu CH, Hwang JK (2006). Prediction of protein subcellular localization. Proteins: Structure, Function, and Bioinformatics.

[ref-45] Zhang G, Chen M, Chen X, Xu Z, Ma Y (2008). Phylogeny, gene structures, and expression patterns of the ERF gene family in soybean (Glycine max L.). Journal of Experimental Botany.

[ref-46] Zhang H, Zhang J, Quan R, Pan X, Wan L, Huang R (2013). EAR motif mutation of rice OsERF3 alters the regulation of ethylene biosynthesis and drought tolerance. Planta.

[ref-47] Zhang ZY, Zhao J, Hu Y, Zhang TZ (2015). Isolation of GhMYB9 gene promoter and characterization of its activity in transgenic cotton. Biologia Plantarum.

[ref-48] Zhao M-J, Yin L-J, Ma J, Zheng J-C, Wang Y-X, Lan J-H, Fu J-D, Chen M, Xu Z-S, Ma Y-Z (2019a). The roles of GmERF135 in improving salt tolerance and decreasing ABA sensitivity in soybean. Frontiers in plant science.

[ref-49] Zhao Y, Ma R, Xu D, Bi H, Xia Z, Peng H (2019b). Genome-wide identification and analysis of the AP2 transcription factor gene family in wheat (Triticum aestivum L.). Frontiers in plant science.

[ref-50] Zhuang J, Cai B, Peng R-H, Zhu B, Jin X-F, Xue Y, Gao F, Fu X-Y, Tian Y-S, Zhao W (2008). Genome-wide analysis of the AP2/ERF gene family in Populus trichocarpa. Biochemical and Biophysical Research Communications.

