# Supplementary material for: Genome-wide identification and expression profiles of ERF subfamily transcription factors in Zea mays
Source: PeerJ. 2020 Jul 17;8:e9551. doi: 10.7717/peerj.9551 (PMC7370932; doi:10.7717/peerj.9551)
Supplement: Table S1 [file peerj-08-9551-s003.pdf]

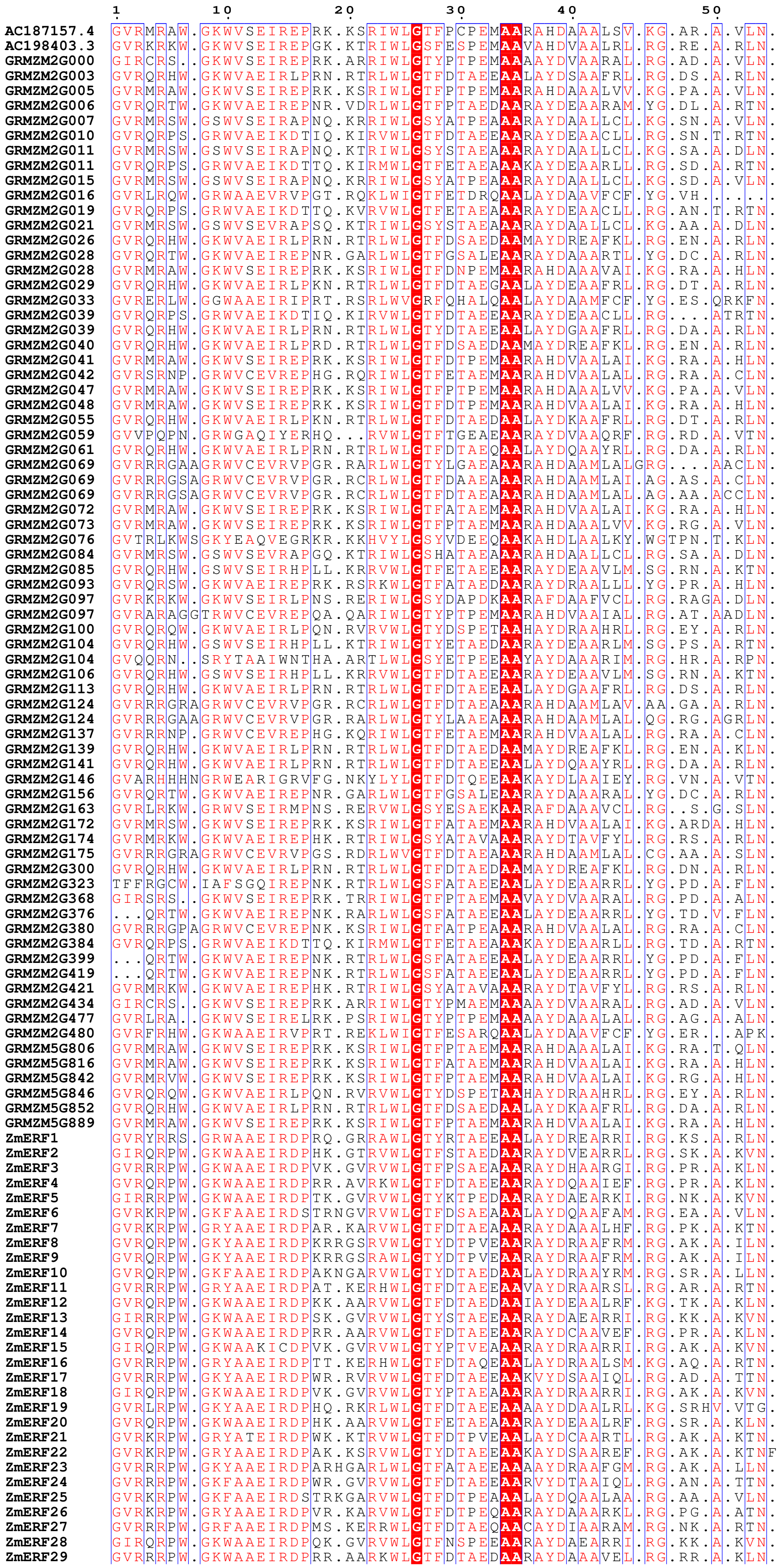
1 10 20 30 40 50
AC187157.4 GVRMRAW.GKWVSEIREPRK.KSRIWLGTFPCPEMAARAHDAAALSV.KG.AR.A.VLN.
AC198403.3 GVRKRKW.GKWVSEIREPGK.KTRIWLGSFESPEMAAVAHDVAALRL.RG.RE.A.RLN.
GRMZM2G000 GIRCRS..GKWVSEIREPRK.ARRIWLGTYPTPEMAAAAYDVAARAL.RG.AD.A.VLN.
GRMZM2G003 GVRQRHW.GKWVAEIRLPRN.RTRLWLGTFDTAEEAALAYDSAAFRL.RG.DS.A.RLN.
GRMZM2G005 GVRMRAW.GKWVSEIREPRK.KSRIWLGTFPTPEMAARAHDAAALVV.KG.PA.A.VLN.
GRMZM2G006 GVRQRTW.GKWVAEIREPNR.VDRLWLGTFPTAEDAARAYDEAARAM.YG.DL.A.RTN.
GRMZM2G007 GVRMRSW.GSWVSEIRAPNQ.KRRIWLGSYATPEAAARAYDAALLCL.KG.SN.A.VLN.
GRMZM2G010 GVRQRPS.GRWVAEIKDTIQ.KIRVWLGTFDTAEEAARAYDEAACLL.RG.SN.T.RTN.
GRMZM2G011 GVRMRSW.GSWVSEIRAPNQ.KTRIWLGSYSTAEAAARAYDAALLCL.KG.SA.A.DLN.
GRMZM2G011 GVRQRPS.GRWVAEIKDTTQ.KIRMWLGTFETAEAAARAYDEAARLL.RG.SD.A.RTN.
GRMZM2G015 GVRMRSW.GSWVSEIRAPNQ.KRRIWLGSYATPEAAARAYDAALLCL.KG.SD.A.VLN.
GRMZM2G016 GVRLRQW.GRWAAEVRVPGT.RQKLWIGTFETDRQAALAYDAAVFCF.YG.VH......
GRMZM2G019 GVRQRPS.GRWVAEIKDTTQ.KVRVWLGTFETAEAAARAYDEAACLL.RG.AN.T.RTN.
GRMZM2G021 GVRMRSW.GSWVSEVRAPSQ.KTRIWLGSYSTAEAAARAYDAALLCL.KG.AA.A.DLN.
GRMZM2G026 GVRQRHW.GKWVAEIRLPRN.RTRLWLGTFDSAEDAAMAYDREAFKL.RG.EN.A.RLN.
GRMZM2G028 GVRQRTW.GKWVAEIREPNR.GARLWLGTFGSALEAARAYDAAARTL.YG.DC.A.RLN.
GRMZM2G028 GVRMRAW.GKWVSEIREPRK.KSRIWLGTFDNPEMAARAHDAAAVAI.KG.RA.A.HLN.
GRMZM2G029 GVRQRHW.GKWVAEIRLPKN.RTRLWLGTFDTAEGAALAYDEAAFRL.RG.DT.A.RLN.
GRMZM2G033 GVRERLW.GGWAAEIRIPRT.RSRLWVGRFQHALQAALAYDAAMFCF.YG.ES.QRKFN.
GRMZM2G039 GVRQRPS.GRWVAEIKDTIQ.KIRVWLGTFDTAEEAARAYDEAACLL.RG....ATRTN.
GRMZM2G039 GVRQRHW.GKWVAEIRLPRN.RTRLWLGTYDTAEEAALAYDGAAFRL.RG.DA.A.RLN.
GRMZM2G040 GVRQRHW.GKWVAEIRLPRD.RTRLWLGTFDSAEDAAMAYDREAFKL.RG.EN.A.RLN.
GRMZM2G041 GVRMRAW.GKWVSEIREPRK.KSRIWLGTFDTPEMAARAHDVAALAI.KG.RA.A.HLN.
GRMZM2G042 GVRSRNP.GRWVCEVREPHG.RQRIWLGTFETAEMAARAHDVAALAL.RG.RA.A.CLN.
GRMZM2G047 GVRMRAW.GKWVSEIREPRK.KSRIWLGTFPTPEMAARAHDAAALVV.KG.PA.A.VLN.
GRMZM2G048 GVRMRAW.GKWVSEIREPRK.KSRIWLGTFDTPEMAARAHDVAALAI.KG.RA.A.HLN.
GRMZM2G055 GVRQRHW.GKWVAEIRLPKN.RTRLWLGTFDTAEDAALAYDKAAFRL.RG.DT.A.RLN.
GRMZM2G059 GVVPQPN.GRWGAQIYERHQ...RVWLGTFTGEAEAARAYDVAAQRF.RG.RD.A.VTN.
GRMZM2G061 GVRQRHW.GKWVAEIRLPRN.RTRLWLGTFDTAEQAALAYDQAAYRL.RG.DA.A.RLN.
GRMZM2G069 GVRRGAAGRWVCEVRVPGR.RARLWLGTYLGEAEAARAHDAAMLALGRG....AACLN.
GRMZM2G069 GVRRGSAGRWVCEVRVPGR.RCRLWLGTFDAAEAAARAHDAAMLAI.AG.AS.A.CLN.
GRMZM2G069 GVRRGSAGRWVCEVRVPGR.RCRLWLGTFDTAEAAARAHDAAMLAL.AG.AA.ACCLN.
GRMZM2G072 GVRMRAW.GKWVSEIREPRK.KSRIWLGTFATAEMAARAHDVAALAI.KG.RA.A.HLN.
GRMZM2G073 GVRMRAW.GKWVSEIREPRK.KSRIWLGTFPTAEMAARAHDAAALVV.KG.RG.A.VLN.
GRMZM2G076 GVTRLKWSGKYEAQVEGRKR.KKHVYLGSYVDEEQAAKAHDLAALKY.WGTPN.T.KLN.
GRMZM2G084 GVRMRSW.GSWVSEVRAPGQ.KTRIWLGSHATAEAAARAHDAALLCL.RG.SA.A.DLN.
GRMZM2G085 GVRQRHW.GSWVSEIRHPLL.KRRVWLGTFETAEEAARAYDEAAVLM.SG.RN.A.KTN.
GRMZM2G093 GVRQRSW.GKWVAEIREPRK.RSRKWLGTFATAEDAARAYDRAALLL.YG.PR.A.HLN.
GRMZM2G097 GVRKRKW.GKWVSEIRLPNS.RERIWLGSYDAPDKAARAFDAAFVCL.RG.RAGA.DLN.
GRMZM2G097 GVRARAGGTRWVCEVREPQA.QARIWLGTYPTPEMAARAHDVAAIAL.RG.AT.AADLN.
GRMZM2G100 GVRQRQW.GKWVAEIRLPQN.RVRVWLGTYDSPETAAHAYDRAAHRL.RG.EY.A.RLN.
GRMZM2G104 GVRQRHW.GSWVSEIRHPLL.KTRIWLGTYETAEDAARAYDEAARLM.SG.PS.A.RTN.
GRMZM2G104 GVQQRN..SRYTAAIWNTHA.ARTLWLGSYETPEEAAYAYDAAARIM.RG.HR.A.RPN.
GRMZM2G106 GVRQRHW.GSWVSEIRHPLL.KRRVWLGTFDTAEEAARAYDEAAVLM.SG.RN.A.KTN.
GRMZM2G113 GVRQRHW.GKWVAEIRLPRN.RTRLWLGTFDTAEEAALAYDGAAFRL.RG.DS.A.RLN.
GRMZM2G124 GVRRGRAGRWVCEVRVPGR.RCRLWLGTFDTAEAAARAHDAAMLAV.AA.GA.A.RLN.
GRMZM2G124 GVRRGAAGRWVCEVRVPGR.RARLWLGTYLAAEAAARAHDAAMLAL.QG.RG.AGRLN.
GRMZM2G137 GVRRNP.GRWVCEVREPHG.KQRIWLGTFETAEMAARAHDVAALAL.RG.RA.A.CLN.
GRMZM2G139 GVRQRHW.GKWVAEIRLPRN.RTRLWLGTFDTAEDAAMAYDREAFKL.RG.EN.A.KLN.
GRMZM2G141 GVRQRHW.GKWVAEIRLPRN.RTRLWLGTFDTAEEAALAYDQAAYRL.RG.DA.A.RLN.
GRMZM2G146 GVARHHHNGRWEARIGRVFG.NKYLYLGTFDTQEEAAKAYDLAAIEY.RG.VN.A.VTN.
GRMZM2G156 GVRQRTW.GKWVAEIREPNR.GARLWLGTFGSALEAARAYDAAARAL.YG.DC.A.RLN.
GRMZM2G163 GVRLRKW.GRWVSEIRMPNS.RERVWLGSYESAEKAARAFDAAAVCL.RG..S.G.SLN.
GRMZM2G172 GVRMRSW.GKWVSEIREPRK.KSRIWLGTFATAEMAARAHDVAALAI.KG.ARDA.HLN.
GRMZM2G174 GVRMRKW.GKWVAEIREPHK.RTRIWLGSYATAVAAARAYDTAVFYL.RG.RS.A.RLN.
GRMZM2G175 GVRRGRAGRWVCEVRVPGS.RDRLWVGTFDTAEAAARAHDAAMLAL.CG.AA.A.SLN.
GRMZM2G300 GVRQRHW.GKWVAEIRLPRN.RTRLWLGTFDTAEDAAMAYDREAFKL.RG.DN.A.RLN.
GRMZM2G323 TFFRGCW.IAFSGQIREPNK.RTRLWLGSFATAEEAALAYDEAARRL.YG.PD.A.FLN.
GRMZM2G368 GIRSRS..GKWVSEIREPRK.TRRIWLGTFPTAEMAAVAYDVAARAL.RG.PD.A.ALN.
GRMZM2G376 ...QRTW.GKWVAEIREPNK.RARLWLGSFATAEEAALAYDEAARRL.YG.TD.V.FLN.
GRMZM2G380 GVRRGPAGRWVCEVREPNK.KSRIWLGTFATPEAAARAHDVAALAL.RG.RA.A.CLN.
GRMZM2G384 GVRQRPS.GRWVAEIKDTTQ.KIRMWLGTFETAEAAAKAYDEAARLL.RG.TD.A.RTN.
GRMZM2G399 ...QRTW.GKWVAEIREPNK.RTRLWLGSFATAEEAALAYDEAARRL.YG.PD.A.FLN.
GRMZM2G419 ...QRTW.GKWVAEIREPNK.RTRLWLGSFATAEEAALAYDEAARRL.YG.PD.A.FLN.
GRMZM2G421 GVRMRKW.GKWVAEIREPHK.RTRIWLGSYATAVAAARAYDTAVFYL.RG.RS.A.RLN.
GRMZM2G434 GIRCRS..GKWVSEIREPRK.ARRIWLGTYPMAEMAAAAYDVAARAL.RG.AD.A.VLN.
GRMZM2G477 GVRLRA..GKWVSEIRELRK.PSRIWLGTYPTPEMAAAAYDAAALAL.RG.AG.A.ALN.
GRMZM2G480 GVRFRHW.GKWAAEIRVPRT.REKLWLGTFESARQAALAYDAAVFCF.YG.ER...APK.
GRMZM5G806 GVRMRAW.GKWVSEIREPRK.KSRIWLGTFPTAEMAARAHDAAALAI.KG.RA.T.QLN.
GRMZM5G816 GVRMRAW.GKWVSEIREPRK.KSRIWLGTFATAEMAARAHDVAALAI.KG.RA.A.HLN.
GRMZM5G842 GVRMRVW.GKWVSEIREPRK.KSRIWLGTFPTAEMAARAHDVAALAI.KG.RG.A.HLN.
GRMZM5G846 GVRQRQW.GKWVAEIRLPQN.RVRVWLGTYDSPETAAHAYDRAAHRL.RG.EY.A.RLN.
GRMZM5G852 GVRQRHW.GKWVAEIRLPRN.RTRLWLGTFDSAEDAALAYDKAAFRL.RG.DA.A.RLN.
GRMZM5G889 GVRMRAW.GKWVSEIREPRK.KSRIWLGTFPTAEMAARAHDVAALAI.KG.RA.A.HLN.
ZmERF1 GVRYRRS.GRWAAEIRDPRQ.GRRAWLGTYRTAEEAALAYDREARRI.RG.KS.A.RLN.
ZmERF2 GIRQRPW.GKWAAEIRDPHK.GTRVWLGTFSTAEDAARAYDVEARRL.RG.SK.A.KVN.
ZmERF3 GVRRRPW.GKWAAEIRDPVK.GVRVWLGTFPSAEAAARAYDHAARGI.RG.PR.A.KLN.
ZmERF4 GVRQRPW.GKWAAEIRDPRR.AVRKWLGTFDTAEEAARAYDQAAIEF.RG.PR.A.KLN.
ZmERF5 GIRRRPW.GKWAAEIRDPTK.GVRVWLGTYKTPEDAARAYDAEARKI.RG.NK.A.KVN.
ZmERF6 GVRKRPW.GKFAAEIRDSTRNGVRVWLGTFDSAEAAALAYDQAAFAM.RG.EA.A.VLN.
ZmERF7 GVRKRPW.GRYAAEIRDPAR.KARVWLGTFDTAEAAARAYDAAALHF.RG.PK.A.KTN.
ZmERF8 GVRQRPW.GKYAAEIRDPKRRGSRVWLGTYDTPVEAARAYDRAAFRM.RG.AK.A.ILN.
ZmERF9 GVRQRPW.GKYAAEIRDPKRRGSRAWLGTYDTPVEAARAYDRAAFRM.RG.AK.A.ILN.
ZmERF10 GVRQRPW.GKFAAEIRDPAKNGARVWLGTYDTAEDAALAYDRAAYRM.RG.SR.A.LLN.
ZmERF11 GVRRRPW.GRYAAEIRDPAT.KERHWLGTFDTAEEAAVAYDRAARSL.RG.AR.A.RTN.
ZmERF12 GVRQRPW.GKWAAEIRDPKK.AARVWLGTFDTAEDAAIAYDEAALRF.KG.TK.A.KLN.
ZmERF13 GIRRRPW.GKWAAEIRDPSK.GVRVWLGTYSTAEEAARAYDAEARRI.RG.KK.A.KVN.
ZmERF14 GVRQRPW.GKWAAEIRDPRR.AARVWLGTFDTAEEAARAYDCAAVEF.RG.PR.A.KLN.
ZmERF15 GIRQRPW.GKWAAKICDPVK.GVRVWLGTYPTVEAAARAYDRAARRI.RG.AK.A.KVN.
ZmERF16 GVRRRPW.GRYAAEIRDPTT.KERHWLGTFDTAQEAALAYDRAALSM.KG.AQ.A.RTN.
ZmERF17 GVRRRPW.GKYAAEIRDPWR.RVRVWLGTFDTAEEAAKVYDSAAIQL.RG.AD.A.TTN.
ZmERF18 GIRQRPW.GKWAAEIRDPVK.GVRVWLGTYPTAEAAARAYDRAARRI.RG.AK.A.KVN.
ZmERF19 GVRLRPW.GKYAAEIRDPHQ.RKRLWLGTFDTAEEAAAAYDDAALRL.KG.SRHV.VTG.
ZmERF20 GVRQRPW.GKWAAEIRDPHK.AARVWLGTFETAEAAARAYDEAALRF.RG.SR.A.KLN.
ZmERF21 GVRKRPW.GRYATEIRDPWK.KTRVWLGTFDTPVEAALAYDCAARTL.RG.AK.A.KTN.
ZmERF22 GVRKRPW.GRYAAEIRDPAK.KSRVWLGTYDTAEEAAKAYDSAAREF.RG.AK.A.KTNF
ZmERF23 GVRRRPW.GKYAAEIRDPARHGARLWLGTFATAEEAAAAYDRAAFGM.RG.AK.A.LLN.
ZmERF24 GVRRRPW.GKFAAEIRDPWR.GVRVWLGTFDTAEEAARVYDTAAIQL.RG.AN.A.TTN.
ZmERF25 GVRKRPW.GKFAAEIRDSTRKGARVWLGTFDTPEAAALAYDQAALAA.RG.AA.A.VLN.
ZmERF26 GVRKRPW.GRYAAEIRDPVR.KARVWLGTFDTPEQAARAYDAAARKL.RG.PG.A.ATN.
ZmERF27 GVRRRPW.GRFAAEIRDPMS.KERRWLGTFDTAEQAACAYDIAARAM.RG.NK.A.RTN.
ZmERF28 GIRQRPW.GKWAAEIRDPQK.GVRVWLGTFNSPEEAARAYDAEARRI.RG.KK.A.KVN.
ZmERF29 GVRRRPW.GKWAAEIRDPRR.AARKWLGTFDTAEDAARAYDAAAVEL.RG.RR.A.KLN.

```
ZmERF30   GVRRRPW.GKYAAEIRDPGIAGKRRWLGTFDTAEEAAAVYDAAALRI.RG.HR.A.VTN.
ZmERF31   GVRQRPW.GKWAAEIRDPHK.AARVWLGTFDTAEAAARAYDVAALRF.RG.SR.A.KLN.
ZmERF32   GVRKRPW.GRYAAEIRDPAK.KARVWLGTFDSAEDAARAYDAAARML.RG.PK.A.RTN.
ZmERF33   GVRRRPS.GRYAAEIRDPAR.KTPIWLGTFESAEAAAHAYDAAARTI.RG.AA.A.RTN.
ZmERF34   GVRKRPW.GRYAAEIRDPAK.KARVWLGTFDSAEDAARAYDAAARML.RG.PK.A.RTN.
ZmERF35   GVRKRPW.GKFAAEIRDSTRKGARVWLGTFDTPEAAALAYDQAALAA.RG.AA.A.VLN.
ZmERF36   GVRRRPW.GKWAAEIRDPAK.AARVWLGTFDTAEAAAAAYDRAALQF.KG.AK.A.KLN.
ZmERF37   GVRRRPW.GRYAAEIRDPAT.KERHWLGTFDTAEEAAIAYDRAARNI.RG.AN.A.RTN.
ZmERF38   GVRQRPW.GKFAAEIRDPAKNGARVWLGTYDSAEDAAVAYDRAAYRM.RG.SR.A.LLN.
ZmERF39   GVRQRPW.GKFAAEIRDPKKRGSRVWLGTYDTAIEAARAYDRAAFRM.RG.AK.A.ILN.
ZmERF40   GVRRRPW.GKWAAEIRDPRR.AARKWLGTFDTAEDAARAYDVAAVEL.RG.QR.A.KLN.
ZmERF41   GIRQRPW.GKWAAEIRDPQK.GVRVWLGTFNSPEEAARAYDAEARRI.RG.KK.A.KVN.
ZmERF42   GVRRRPW.GKYAAEIRDPHK.GERLWLGTFDTAEEAAREYDSAARRL.RG.PS.A.TTN.
ZmERF43   GVRRRPS.GRYAAEIRDPAK.KTPIWLGTFDSAEAAARAYDAAARSL.RG.PT.A.RTN.
ZmERF44   GIRRRPW.GRWAAEIRDPRK.GARVWLGTYATPEDAARAYDVAAREI.RG.PK.A.KLN.
ZmERF45   GVRQRPW.GKWAAEIRDPHK.AARVWLGTFETAEAAARAYDEAALRF.RG.SR.A.KLN.
ZmERF46   GVRKRPW.GRYAAEIRDPWR.KTRVWLGTYDTPVDAAMAYDRAAVAL.RG.AK.A.RTN.
ZmERF47   GVRKRPW.GRYAAEIRDPAK.KSRVWLGTFDTPEDAARAYDAAAREY.RG.AK.A.KTNF
ZmERF48   GVRRRPW.GKFAAEIRDTRRRGARVWLGTFATADDAALAYDKAALRM.RG.PR.A.CLN.
ZmERF49   GVRRRPW.GKFAAEIRDSTRNGQRVWIGTFDTPEAAALAYDQAAYSM.RS.TA.A.VLN.
ZmERF50   GVRRRPW.GKFAAEIRDSTRNGERVWIGTFDTPEAAALAYDQAAYSM.RS.SS.A.VLN.
ZmERF51   GVRRRPW.GRWAAEIRDPML.RRRVWLGTFDTAEEAAAAYVAARLRI.RG.PK.A.SGSN
ZmERF52   GVRRRPW.GRFAAEIRDSTRNGARVWLGTFDSAEAAAMAYDQAALSA.RG.SA.A.ALN.
ZmERF53   GVRKRPW.GKFAAEIRDSTRKGARVWLGTFDSPEAAAMAYDQAAFSV.RG.AA.A.VLN.
ZmERF54   GVRRRRW.GRWAAEIRDPVR.KTRKWIGSYDSEEAAAAAYQAYAKQI.RE.EL.L.AIK.
ZmERF55   GIRQRPW.GKWAAEIRDPNK.GVRVWLGTYNTAEEAARAYDAEARKI.RG.KK.A.KVN.
ZmERF56   GVRRRPW.GRWAAEIRDPEK.AARVWLGTFATPEEAARAYDDAARRF.KG.AK.A.KLN.
ZmERF57   GVRRRPW.GRYAAEIRDPTT.KERHWLGTFDTAQEAALAYDRAALSM.KG.AQ.A.RTN.
ZmERF58   GIRRRPW.GKWAAEIRDPAK.GARVWLGTFATAEAAARAYDRAARRI.RG.AK.A.KVN.
ZmERF59   GVRRRPW.GKYAAEIRDPWR.RVRVWLGTFDTAEEAAKVYDSAAVQL.RG.RD.A.TTN.
ZmERF60   GIRQRPW.GKWAAEIRDPVK.GVRVWLGTYPTAEAAARAYDRAARRI.RG.AK.A.KVN.
ZmERF61   GVRRRPW.GKFAAEIRDTRRKGARVWLGTFATAEDAALAYDKAALRM.RG.PR.A.HLN.
ZmERF62   GVRRRPW.GRFAAEIRDPMS.KERRWLGTFDTAEQAACAYDIAARAM.RG.NK.A.RTN.
ZmERF63   GVRQRPW.GKWAAEIRDPHK.AARVWLGTFDTAEAAARAYDEAALRF.RG.SR.A.KLN.
ZmERF64   GVRKRPW.GRYAAEIRDPWR.KTRVWLGTYDTPVEAAMAYDRAAVAL.RG.SK.A.RTN.
ZmERF65   GVRKRPW.GRYAAEIRDPAK.KSRVWLGTYDTAEDAARAYDAAAREY.RG.AK.A.KTN.
ZmERF66   GVRRRPW.GKFAAEIRDPWR.GVRVWLGTFDTAEEAARVYDAAAVQL.RG.AN.A.TTN.
ZmERF67   GVRKRPW.GRYAAEIRDPAK.KSRVWLGTYDTAEEAAKAYDVAAREF.RG.AK.A.KTNF
ZmERF68   GIRRRPW.GKWAAEIRDPRK.GVRVWLGTFNSPEEAARAYDAEARRI.RG.KK.A.KVN.
ZmERF69   GVRRRPW.GRYAAEIRDPHK.GERLWLGTFDTAEEAARRYDSETRRL.RG.PS.A.ITN.
ZmERF70   GIRRRPW.GKWAAEIRDPSK.GVRVWLGTYSTAEEAARAYDAEARRI.RG.KK.A.KVN.
ZmERF71   GVRQRPW.GKWAAEIRDPHK.AARVWLGTFDSAEAAARAYDGAALRF.RG.SR.A.KLN.
ZmERF72   GVRQRPW.GKWAAEIRDPKK.AARVWLGTFDTAEDAAIAYDEAALRF.KG.TK.A.KLN.
ZmERF73   GVRRRPW.GRYAAEIRDPAT.KERHWLGTFDTAEEAAVAYDRAARSL.RG.AR.A.RTN.
ZmERF74   GVRRRPW.GKWAAEIRDPQK.AARVWLGTFPTAEDAARAYDAAALRF.RG.GR.A.KLN.
ZmERF75   GVRQRPW.GKFAAEIRDPARNGARVWLGTYDTAEDAALAYDRAAYRM.RG.SR.A.LLN.
ZmERF76   GVRKRPW.GRFAAEIRDPAR.KARVWLGTFDTAEAAARAYDAAALHF.RG.PK.A.KTN.
```
