# Supplementary figures and images for: Genome-wide identification and expression profiles of ERF subfamily transcription factors in Zea mays
Source: PeerJ. 2020 Jul 17;8:e9551. doi: 10.7717/peerj.9551 (PMC7370932; doi:10.7717/peerj.9551)

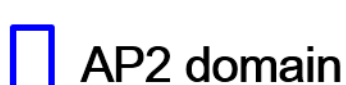

Supplement: Figure S2 — There is a clear AAEIRD-motif in the AP2 domain. [file peerj-08-9551-s004.pdf]

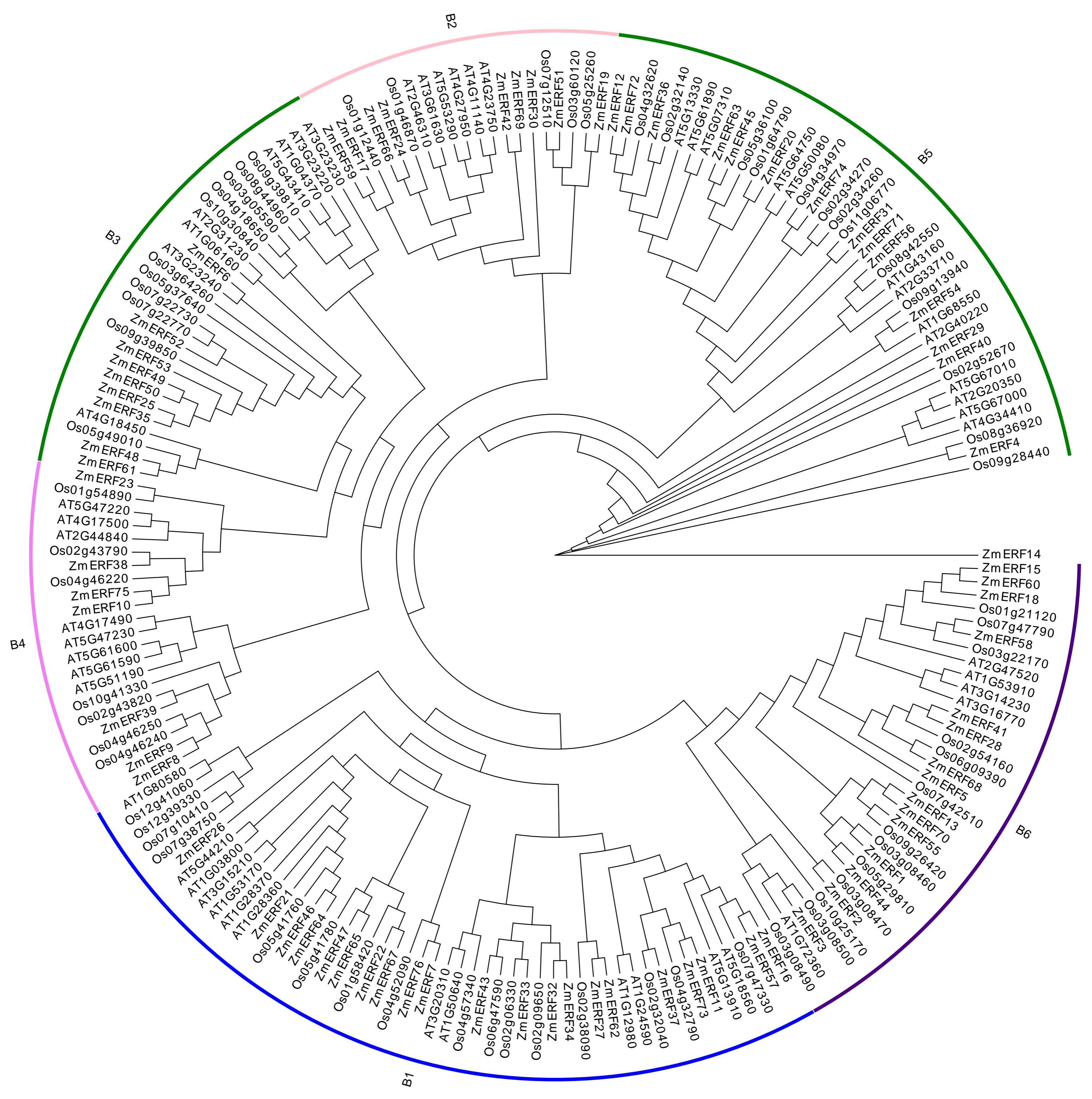

Supplement: Figure S3 [file peerj-08-9551-s005.pdf]
